# Causal role of immune cell phenotypes in idiopathic sudden sensorineural hearing loss: a bi-directional Mendelian randomization study

**DOI:** 10.3389/fneur.2024.1368002

**Published:** 2024-04-17

**Authors:** Wanqing Li, Qiang Zhou, Linsa Zhou, Longhe Cao, Chuansai Zhu, Zhijian Dai, Sen Lin

**Affiliations:** ^1^Department of Otolaryngology, Ruian People’s Hospital, The Third Affiliated Hospital of Wenzhou Medical University, Wenzhou, China; ^2^Department of Burns and Plastic Surgery, The Second Affiliated Hospital of Shantou University Medical College, Shantou, China

**Keywords:** idiopathic sudden sensorineural hearing loss, immunophenotypes, Mendelian randomization, causality, sensitivity analysis

## Abstract

**Background:**

A growing body of evidence suggests that immunological processes have a significant role in developing idiopathic sudden sensorineural hearing loss (SSHL). However, few studies have examined the association between immune cell phenotype and SSHL using Mendelian Randomization (MR).

**Methods:**

The online genome-wide association studies (GWAS) database was used to compile data from GWAS covering 731 immunophenotypes and SSHL. Inverse variance weighted (IVW) analysis was primarily used for MR study, and single nucleotide polymorphisms (SNPs) associated with immunophenotypes served as dependent variables. A sensitivity study and the false discovery rate (FDR) correction were used to examine the MR hypothesis. In addition, the possibility of reverse causality between immunophenotype and SSHL was validated by reverse MR. Reverse MR was analyzed in a manner consistent with forward MR.

**Results:**

After FDR correction and sensitivity analysis, we screened 7 immunophenotypes, including IgD^+^ CD38^dim^ %lymphocyte (95% CI: 1.0019, 1.0742, *p* = 3.87 × 10^−2^, FDR = 1.15 × 10^−2^); Unsw mem AC (95% CI: 1.004, 1.2522, *p* = 4.23 × 10^−2^, FDR = 2.25 × 10^−2^); CD86^+^ myeloid DC AC (95% CI: 1.0083, 1.1147, *p* = 2.24 × 10^−2^, FDR = 4.27 × 10^−2^); CD33^dim^ HLA DR^−^ AC (95% CI: 1.0046, 1.0583, *p* = 2.12 × 10^−2^, FDR = 4.69 × 10^−2^); SSC-A on CD8^br^ (95% CI: 1.0028, 1.1461, *p* = 4.12 × 10^−2^, FDR = 4.71 × 10^−2^); CD45RA^−^ CD4^+^ %T cell (95% CI: 1.0036, 1.0503, *p* = 2.32 × 10^−2^, FDR = 4.82 × 10^−2^); DP (CD4^+^CD8^+^) AC (95% CI: 1.011, 1.2091, *p* = 2.78 × 10^−2^, FDR = 4.97 × 10^−2^). There was a strong causal relationship with SSHL onset, and the reliability of the results was verified. Furthermore, the immunological cell profile and SSHL did not appear to be closely associated, as shown by reverse MR analysis.

**Conclusion:**

Our study provides more support for the current hypothesis that immunophenotypes and the pathophysiology of SSHL are closely associated. Further validation is needed to assess the role of these immunophenotypes in SSHL.

## Introduction

The National Institute on Deafness and Other Communication Disorders defines idiopathic sudden sensorineural hearing loss (SSHL), as a condition distinguished by an abrupt and inexplicable decrease in hearing of at least 30 decibels at 3 subsequent sounds (less than 3 dB) without a discernible cause (1). The loss of hearing usually involves only one side of the ear, with less than 2% of cases involving both ears. It might appear suddenly or within a few hours (2). A German study estimated the prevalence rate to be 160 instances per 100,000 individuals annually (3).

While the incidence of SSHL increases with age, there are no significant gender differences (4). SSHL is considered a medical emergency, and its evolution is variable and multifactorial, with limited relevant studies available (5). However, the impact on the patient should not be underestimated. Individuals diagnosed with SSHL often develop acute auditory losses that worsen rapidly or unexpectedly, and there is often a delay in seeking medical attention (3). Since it is not feasible to biopsy and pathologically analyze the inner ear *in vivo*, studies on the etiology of SSHL can only be conducted by analyzing data obtained from peripheral blood or imaging studies. Despite the large number of studies on SSHL, research into its pathogenesis remains limited (6). Though many individuals lack an apparent cause SSHL, abrupt deafness can be linked to infections, vascular injuries, autoimmune diseases, injury, internal ear anomalies, and neurological diseases (7). An increasing amount of research indicates that immune-associated processes may play essential role in deafness as well as mechanisms involving autoimmune and autoinflammatory diseases may also affect hearing. For example, autoimmune inner ear disease (8), Meniere’s disease (9), Cogan’s syndrome (10), and NLRP3-related autoinflammatory diseases (11) have been found to be strongly associated with SSHL (12). Research findings indicate that elevated antibody levels may lead to the condition through indirect reactions with internal hearing antigens or stimulated T-cells. Autoantibodies against collagen types 2 and 9 and other internal ear antigens were also identified in SSHL patients (13) Regarding the treatment of SSHL, oral corticosteroids are widely recognized as playing a primary role in suppressing the immune reaction (14). Nevertheless, the pathology of immune-mediated sensorineural hearing loss remains unclear (15). Thus, the purpose of our study was to determine that immune cell morphologies and SSHL are directly associated. By employing a Mendelian Randomization (MR) approach, we can minimize confounders and eliminate reverse causality. In the present study, we utilized genetic variants strongly linked to immunocyte phenotypes and SSHL as instrumental variables. This is a significant advancement in the comprehension of the direct mechanistic link between immunological cells and SSHL. This study provides more concrete evidence that the pathogenesis of SSHL may be linked to immunity, offering new perspectives on the diagnosis, treatment, and research direction of SSHL.

## Methods

### Research design

**Figure 1:**
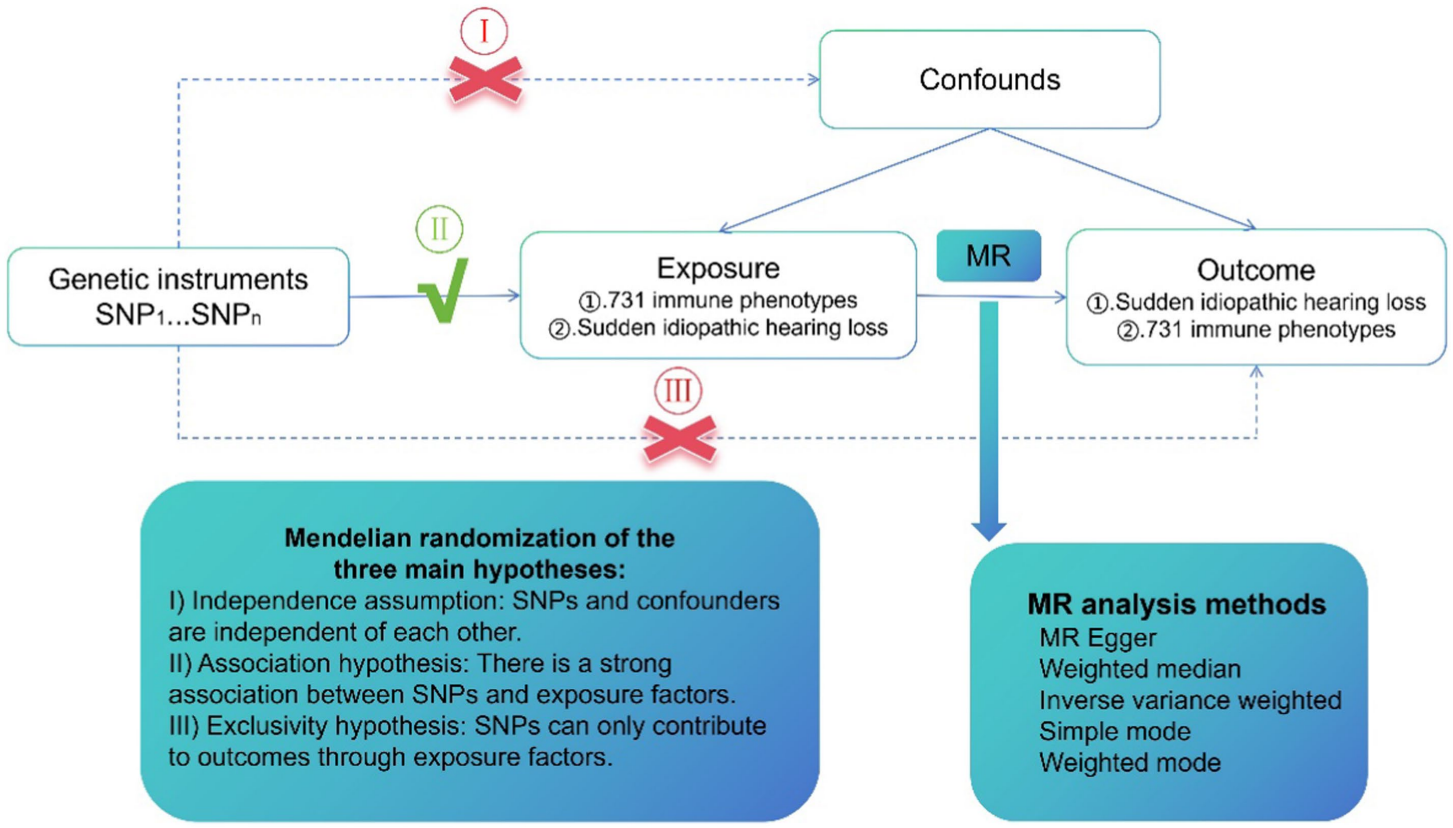
A design scheme for Mendelian randomization (MR). Forward MR: the exposure factor is the immune phenotypes and the outcome factor is Idiopathic sudden sensorineural hearing loss (SSHL). Reverse MR: the exposure factor is SSHL and the end factor is immune phenotypes. Single nucleotide polymorphisms (SNPs) stands for single nucleotide diversity.

The possible association of 731 immunophenotypes and SSHL has been assessed using single-nucleotide polymorphisms (SNPs), which are considered instrumental variables (IVs) derived from extensive GWAS studies. The study design employed a Bi-directional Mendelian randomization (MR) analysis method and the SNPs required three key assumptions, as shown in Figure 1. The MR method relies on three main hypotheses. Firstly, the independence assumption states that SNPs and confounders are mutually independent. Secondly, the association hypothesis suggests a strong association between SNPs and exposure factors. Finally, the exclusivity hypothesis states that SNPs can only influence outcomes through exposure factors. Participant’s written authorization was obtained by confirming consent forms, and the data utilized in this investigation had been authorized by the appropriate ethical evaluation boards.

### Availability of SSHL genome-wide association study (GWAS) data

The GWAS ID: finn-b-H8_HL_IDIOP associated with SSHL obtained the summary-level data using the IEU Open GWAS program (https://gwas.mrcieu.ac.uk/datasets/finn-b-H8_HL_IDIOP/). Following evaluation and estimation, the dataset includes 196,592 instances and 1,491 controls with European ancestry, including 16,380,424 variations (16).

### Collection of the GWAS data across immunity

The publically available GWAS database has been the source of all immunophenotypes information. We selected immunophenotypes-related data with numbers between GCST0001391 and GCST0002121 (17). The research included a broad spectrum of 731 immunophenotypes, including 118 relative cell counts (RC), 32 morphologic parameters (MP), 389 median fluorescence intensity (MFI) values indicating surface antigen levels, and 118 absolute cell counts (AC).

The immunophenotype dataset contained parameters such as MFI, AC, and RC that collected data on B-cells, Treg panels, cDCs, T-cell activation phase, monocytes, myeloid cell types, and TBNK (T-cells, B-cells, and natural killer cells). The TBNK and cDCs panels were included in the MP feature. There were 3,757 Europeans, that were involved in the initial GWAS for immune-mediated modeling, did not belong to any overlapped groups. Correlation analyses were run on the nearly 22,000,000 SNPs that were identified using high-density arrays whenever traits including gender, age, and age square were taken into consideration. Supplementary file 1 and the website at https://www.ebi.ac.uk/gwas/ provide access for the complete information.

### IVs selection

Suitable IVs from the different GWAS results were obtained separately for MR analysis. For immune cells, we screened for SNPs in the European 1,000 Genomes reference group using a *p*-value less than 1 × 10^−5^ as a statistical criterion and linking disequilibrium elimination (the screening condition was *r*^2^ < 0.001, within 10,000 kb). We selected *F* < 10 to represent the reliability of the weak instrument and then assessed the *F*-value after adjusting its significance level to 5 × 10^−8^.

### MR analysis

To thoroughly evaluate the causal relationship between 731 immune phenotypes and SSHL, we conducted forward MR analysis with the 731 immune phenotypes as the exposure factors and reverse MR analysis with SSHL as the exposure factor to enhance the reliability of the causal inference and eliminate confounding factors. We employed the inverse-variance weighted (IVW) method (18), median-based weighted method (19), and mode-based weighted method (20), with the IVW model serving as our primary analysis approach as it is widely recognized as a robust method in MR studies. Subsequently, Cochran’s Q statistic (21) and the chosen IVs were examined for overall heterogeneity by a compatible *p*-value. Furthermore, we utilized the “leave-one-out” approach (22) to assess the robustness of the MR results and identify sources of heterogeneity. Finally, we used the powerful tool MR-PRESSO (23) to eliminate the consequences of pleiotropy on the horizon, remove outlier SNPs, estimate corrected results, and test for differences between pre- and post-corrected results.

### Statistical analysis

The R 4.3.1 program was used to perform the statistical analysis (http://www.Rproject.org). The “TwoSampleMR” program (24) was the primary tool used to assess the probable association across 731 immunophenotypes and SSHL. The findings were considered trustworthy when an FDR modification (FDR < 0.05) had been applied to control for various comparisons and a threshold value of *p* < 0.05 was achieved.

## Results

### Forward MR analysis: immunophenotypes on SSHL

**Figure 2:**
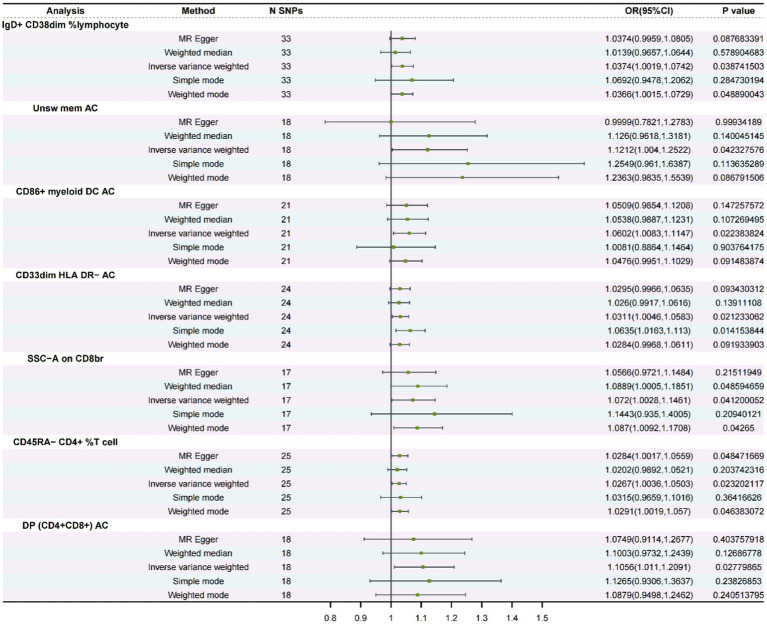
A forest diagram involving five approaches to analyze the association between immunological phenotypes and SSHL (Forward MR).

To find a possible link between susceptibility and 731 immune-mediated immunophenotypes, we conducted a bidirectional MR investigation in the present study. We identified seven immune phenotypes associated with SSHL using mainly the IVW method and using FDR correction (FDR < 0.05) as a criterion for correlation (Supplementary file 2: Table S1). Subsequently, we assessed the stability of the results by sensitivity analyses, which revealed that seven immunophenotypes were associated with SSHL (Figure 2; Supplementary file 2: Table S2). The screening program identified two panel for B cells (IgD^+^ CD38^dim^ % lymphocyte and Unsw mem AC), one panel for conventional Dendritic Cells (cDCs) identified as CD86^+^ myeloid DC AC, one panel for myeloid cells (CD33^dim^ HLA DR^−^ AC), two panels for detecting TBNKs (T cells, B cells, and NK cells) which included SSC-A on CD8^br^ and DP (CD4^+^CD8^+^) AC, and one panel is for the maturation stage of T cells (CD45RA^−^ CD4^+^ %T cells). Utilizing the IVW method, we obtained an odds ratio (OR) for SSHL risk of 1.0374 (95% CI: 1.0019–1.0742, *p* = 3.87 × 10^−2^, FDR = 1.15 × 10^−2^) for IgD^+^ CD38^dim^ % lymphocyte. Weighted mode and weighted median methods were also employed, resulting in odds ratios of 1.0366 (95% CI: 1.0015–1.0729, *p* = 4.89 × 10^−2^) and 1.0139 (95% CI: 0.9657–1.0644, *p* = 5.79 × 10^−1^). By the IVW method, we obtained an outcome Unsw mem AC odds ratio (OR) for SSHL risk of 1.1212 (95% CI: 1.004–1.2522, *p* = 4.23 × 10^−2^, FDR = 2.25 × 10^−2^). And it was obtained by weighted mode method (OR = 1.2363, 95% CI: 0.9835–1.5539, *p* = 8.68 × 10^−2^) and weighted median method (OR = 1.126, 95% CI: 0.9618–1.3181, *p* = 1.40 × 10^−1^). By the IVW method, we obtained an outcome CD86^+^ myeloid DC AC odds ratio (OR) for SSHL risk of 1.0602 (95% CI: 1.0083–1.1147, *p* = 2.24 × 10^−2^, FDR = 4.27 × 10^−2^). And it was obtained by weighted mode method (OR = 1.0476, 95% CI: 0.995–1.1029, *p* = 9.15 × 10^−2^) and weighted median method (OR = 1.0538, 95% CI: 0.9887–1.1231, *p* = 1.07 × 10^−1^). By the IVW method, we obtained an outcome CD33^dim^ HLA DR^−^ AC odds ratio (OR) for SSHL risk of 1.0311 (95% CI: 1.0046–1.0583, *p* = 2.12 × 10^−2^, FDR =4.69 × 10^−2^). And it was obtained by weighted mode method (OR = 1.0284, 95% CI: 0.9968–1.0611, *p* = 9.19 × 10^−2^) and weighted median method (OR = 1.026, 95% CI: 0.9917–1.0616, *p* = 1.39 × 10^−1^). By the IVW method, we obtained an outcome SSC-A on CD8^br^ odds ratio (OR) for SSHL risk of 1.072 (95% CI: 1.0028–1.1461, *p* = 4.12 × 10^−2^, FDR =4.71 × 10^−2^). And it was obtained by weighted mode method (OR = 1.087, 95% CI: 1.0092–1.1708, *p* = 4.27 × 10^−2^) and weighted median method (OR = 1.0889, 95% CI: 1.0005–1.1851, *p* = 4.86 × 10^−2^). By the IVW method, we obtained an outcome CD45RA^−^ CD4^+^ %T cell odds ratio (OR) for SSHL risk of 1.0267 (95% CI: 1.0036–1.0503, *p* = 2.32 × 10^−2^, FDR =4.82 × 10^−2^). And it was obtained by weighted mode method (OR = 1.0291, 95% CI: 1.0019–1.057, *p* = 4.64 × 10^−2^) and weighted median method (OR = 1.0202, 95% CI: 0.9892–1.0521, *p* = 2.04 × 10^−1^). Finally, by the IVW method, we obtained an outcome DP (CD4^+^CD8^+^) AC odds ratio (OR) for SSHL risk of 1.1056 (95% CI: 1.011–1.2091, *p* = 2.78 × 10^−2^, FDR =4.97 × 10^−2^). And it was obtained by weighted mode method (OR = 1.0879, 95% CI: 0.9498–1.2462, *p* = 2.41 × 10^−1^) and weighted median method (OR = 1.1003, 95% CI: 0.9732–1.2439, *p* = 1.27 × 10^−1^).

Furthermore, for the seven immunophenotypes examined, the MR-Egger’s intercepting analysis and the MR-PRESSO worldwide testing showed no horizontal pleiotropy (Supplementary file 2: Table S3). Subsequently, Cochran’s Q test and “leave-one-out” screening were performed, wherein none of the obtained results displayed any heterogeneity (Table 1 and Supplementary Figure S1). Scatter plots, funnel plots and forest plots serve to accentuate the rigor of the outcomes (Supplementary Figures S2–S4).

**Table 1:** The Cochran’s *Q* test of inverse variance weighting (IVW) and MR-Egger (Forward MR).

id.exposure	id.outcome	outcome	exposure	method	Q	Q_df	Q_pval
ebi-a-GCST90001430	0d6IHi	SSHL	IgD^+^ CD38^dim^ %lymphocyte	MR Egger	37.57413169	31	0.193336613
ebi-a-GCST90001430	0d6IHi	SSHL	IgD^+^ CD38^dim^ %lymphocyte	Inverse variance weighted	37.57417349	32	0.228953181
ebi-a-GCST90001398	D3bYLG	SSHL	Unsw mem AC	MR Egger	18.38088416	16	0.302069657
ebi-a-GCST90001398	D3bYLG	SSHL	Unsw mem AC	Inverse variance weighted	19.58308913	17	0.296106803
ebi-a-GCST90001464	M75iEr	SSHL	CD86^+^ myeloid DC AC	MR Egger	22.94188856	19	0.239911053
ebi-a-GCST90001464	M75iEr	SSHL	CD86^+^ myeloid DC AC	Inverse variance weighted	23.17638993	20	0.280216657
ebi-a-GCST90001531	MVDw59	SSHL	CD33^dim^ HLA DR- AC	MR Egger	11.78842616	22	0.961595725
ebi-a-GCST90001531	MVDw59	SSHL	CD33^dim^ HLA DR- AC	Inverse variance weighted	11.81216066	23	0.973267479
ebi-a-GCST90002082	99MAuu	SSHL	SSC-A on CD8^br^	MR Egger	17.48096332	15	0.290934211
ebi-a-GCST90002082	99MAuu	SSHL	SSC-A on CD8^br^	Inverse variance weighted	17.89171786	16	0.330275783
ebi-a-GCST90001536	uchPZN	SSHL	CD45RA^−^ CD4^+^ %T cell	MR Egger	23.5592922	23	0.428536994
ebi-a-GCST90001536	uchPZN	SSHL	CD45RA^−^ CD4^+^ %T cell	Inverse variance weighted	23.62642749	24	0.48311714
ebi-a-GCST90001594	ZuD9Gw	SSHL	DP (CD4^+^CD8^+^) AC	MR Egger	8.221609321	16	0.941993345
ebi-a-GCST90001594	ZuD9Gw	SSHL	DP (CD4^+^CD8^+^) AC	Inverse variance weighted	8.380641727	17	0.957716303

### Reverse MR analysis: SSHL on immunophenotypes

We combined seven immunocyte phenotypes obtained from forward MR analyses: IgD^+^ CD38^dim^ % lymphocytes and Unsw mem AC, CD86^+^ myeloid DC AC, CD33^dim^ HLA DR^−^ AC, SSC-A on CD8^br^ and DP (CD4^+^CD8^+^) AC, and CD45RA^−^ CD4^+^ % T cells, and used them as exposure factors. To investigate the potential direct association between the two, SSHL was employed as an outcome factor. Moreover, utilizing 1 × 10^−5^ as the threshold screening condition yielded a total of 23 SNPs. The reverse MR approach’s findings are displayed in Table 2, indicating that there was no causal association between SSHL and these seven immunophenotypes.

**Table 2:** The causal relationships between SSHL and seven immune cell phenotypes analyzed by five methods (Reverse MR).

Analysis	Method	N snp	Pval	OR	Lower_95%CI	UP_95%CI
Unsw mem AC						
	MR Egger	22	0.452884933	9.79E-1	-7.45E-2	3.26E-2
	Weighted median	22	0.344504726	9.74E-1	−8.19E-2	2.86E-2
	Inverse variance weighted	22	0.108288949	9.70E-1	−6.68E-2	6.64E-3
	Simple mode	22	0.314807017	9.54E−1	-1.37E-1	4.27E-2
	Weighted mode	22	0.319685255	9.73E-1	−8.04E-2	2.54E-2
IgD^+^ CD38^dim^ %lymphocyte						
	MR Egger	22	0.548764347	1.02	−3.65E−2	6.96E-2
	Weighted median	22	0.348824609	1.02	-2.62E-2	7.45E-2
	Inverse variance weighted	22	0.063190483	1.04	−1.900E-3	7.1E−2
	Simple mode	22	0.214428134	1.06	-2.89E-2	1.38E−1
	Weighted mode	22	0.234160463	1.03	-1.77E−2	7.65E-2
CD86^+^ myeloid DC AC						
	MR Egger	22	0.313144297	1.03	-2.76E-2	8.92 E-2
	Weighted median	22	0.494421898	1.02	−3.62E-2	7.49 E-2
	Inverse variance weighted	22	0.975914687	1.00	−3.92E-2	4.04 E-2
	Simple mode	22	0.920329037	1.00	−7.74E-2	8.58 E-2
	Weighted mode	22	0.450834534	1.02	−3.08E-2	7.04 E-2
CD33^dim^ HLA DR^−^ AC						
	MR Egger	22	0.76178621	9.88E-1	−8.57E-2	6.24E-2
	Weighted median	22	0.723403386	9.87E-1	−8.76E-2	6.08E-2
	Inverse variance weighted	22	0.928628444	1.00	−4.84E-2	5.30E-2
	Simple mode	22	0.349032413	9.48E−1	-1.64E-1	5.63E-2
	Weighted mode	22	0.738260796	9.88E-1	−8.05E-2	5.68E-2
CD45RA^−^ CD4^+^ %T cell						
	MR Egger	22	0.381561674	9.74E-1	−8.27E-2	3.09E-2
	Weighted median	22	0.488062068	9.79E-1	−8.03E-2	3.83E-2
	Inverse variance weighted	22	0.286896943	9.80E-1	−5.85E-2	1.73E-2
	Simple mode	22	0.197342872	9.32E−1	-1.74E-1	3.33E-2
	Weighted mode	22	0.923568877	1.00	−5.58E-2	6.16E-2
DP (CD4^+^CD8^+^) AC						
	MR Egger	22	0.749928383	9.90E-1	−7.37E-2	5.28E-2
	Weighted median	22	0.515868354	9.80E-1	−8.05E-2	4.04E-2
	Inverse variance weighted	22	0.784093794	9.94E-1	−4.81E-2	3.63E-2
	Simple mode	22	0.578019013	9.72E−1	-1.26E-1	6.97E-2
	Weighted mode	22	0.584995428	9.86E-1	−6.48E-2	3.62E-2
SSC-A on CD8^br^						
	MR Egger	22	0.748939761	1.01	−4.44E-2	6.21E-2
	Weighted median	22	0.643553447	9.88E-1	−6.30E-2	3.89E-2
	Inverse variance weighted	22	0.682656057	9.92E-1	−4.39E-2	2.88E-2
	Simple mode	22	0.53454954	9.73E−1	-1.14E-1	5.82E-2
	Weighted mode	22	0.918472063	1.00E-1	−4.47E-2	4.96E-2

## Discussion

In the quest to unravel the complex web of immunocyte phenotypes contributing to Idiopathic sudden sensorineural hearing loss (SSHL), establishing causality remains a significant challenge. Traditional observational studies, while invaluable, often mired by confounding factors and reverse causation, limiting their ability to infer causal relationships. Mendelian Randomization (MR), leveraging gene variations as auxiliary factors, offers a robust alternative by capitalizing on the random assortment of alleles at conception to mimic randomized controlled trials. The Bidirectional MR method was used in the present study to examine the mechanistic interaction between SSHL and 731 immunocyte phenotypes, and results were validated by sensitivity analyses showing that 7 cell immunocyte phenotype as danger factors for SSHL. Additionally, we performed reverse MR with immunophenotype as the final phenotype and SSHL as a risk factor. The results demonstrated that there was no significant association between the two, which further supported the validity of the findings. The study revealed that the proportion of IgD^+^ CD38^dim^% lymphocytes raised the probability of developing SSHL. The role of IgD, though less elucidated compared to other immunoglobulins, is thought to influence B cell activity modulation. On the B cell surface, IgD participates in the primary immune response along with IgM as part of the B cell receptor (BCR), and is closely associated with immunity-related diseases. Additionally, the expression level of CD38 reflects the activation status and maturity of B cell. This result aligns with multiple current case reports where patients with B-cell abnormalities were diagnosed with SSHL. (25, 26) Among them, R-CHOP treatment led to remission in three cases, while two patients died during chemotherapy. One hypothesis suggests that SSHL in these patients may be related to a labyrinthine infarct caused by the lymphoma accumulation (27).

Based on MR data, SSC − A on CD8^br^ and CD45RA^−^ CD4^+^ %T cell were both linked to an increased probability of SSHL. There is growing evidence of the involvement of the immune system, particularly T-lymphocytes and specific autoimmune reactive antibodies (28). Humoral and cell-driven responses cause susceptible antigen-presenting cells (B and T-cells) to produce autoantibodies toward tissues involved in the auditory pathway when interleukin-17, interferon-gamma, and tumor necrosis factor are expressed (12). Ben-Sasson SZ et al. confirmed that the cytokine IL-1 can enhance the antigen-driven response of CD4 and CD8 T cells (29). Additionally, in the presence of IL-1, monocytes produce autoantibodies that damage hearing organs and trigger an acute inflammatory episode (30). Our Mendelian randomization (MR) results support this finding, indicating a genetic association between CD4 and CD8 T cells and SSHL. Several immunomodulators have been explored for their potential use in immune-mediated hearing loss. These include anakinra (IL-1 inhibitor), canakinumab (IL-1 inhibitor), tocilizumab (IL-6 inhibitor), infliximab (TNF-alpha inhibitor), and rituximab (B-cell inhibitor), all of which have displayed efficacy. Intriguingly, Zhou et al. presented a MR analysis (31) indicating that C-reactive protein is a risk factor for SSHL, while TNF-α and fibrinogen do not increase the risk for SSHL. Therefore, TNF-α, an inflammatory marker, exhibited elevated levels in SSHL, but without any relation to its progression.

In addition, MR results showed that Unsw mem AC, CD86^+^ myeloid DC AC, CD33d^im^ HLA DR^−^ AC and DP (CD4^+^CD8^+^) AC were linked to an increased probability of SSHL. Professional APC, such as dendritic cell, monocyte/macrophage and B-cell, detect foreign pathogens through specialized receptors known as pattern recognition receptors (PRRs), making them crucial components of the immune system. Despite the importance of APCs in immune responses, research on their role in SSHL is currently limited. However, Dichhoeck et al. (32) reported that 40 out of 100 patients had respiratory infections associated with SSHL. In addition, Seltzer and Mark (33) revealed the enhancement of magnetic resonance imaging (MRI) in the interior ear of SSHL sufferers indicates the existence of persistent inflammation. The results support the strong correlation between autoimmune and SSHL. However, several limitations must be considered when interpreting our findings. Firstly, although MR is more resistant to unmeasured confounders than traditional epidemiological methods, our results may still be affected by unobserved environmental and physiological factors. In addition, SSHL encompasses various types based on the frequency and degree of hearing loss, including high-frequency descending, low-frequency descending, flat descending, and total deafness. Since we used summary statistics rather than raw data, detailed analysis of these subgroups was not feasible. Furthermore, we were unable to obtain specific treatment information for each patient from the FinnGen database we used making our conclusions somewhat limited. Finally, it is challenging to generalize our findings to other ethnic populations because the study’s findings only cover Europeans.

## Conclusion

We obtained a substantial number of study samples and utilized IVs as a tool to mitigate the influence of confounding factors. Our comprehensive Bidirectional MR analysis demonstrated a significant correlation between 7 immune phenotypes and SSHL. This scientifically rigorous approach minimizes the impact of reverse causality, and the findings may open new avenues for research and advancements in the diagnosis, treatment, and intervention of SSHL.

## Data availability statement

The original contributions presented in the study are included in the article/Supplementary material, further inquiries can be directed to the corresponding authors.

## Author contributions

WL: Conceptualization, Data curation, Formal analysis, Methodology, Software, Validation, Visualization, Writing – original draft, Writing – review & editing, Supervision. QZ: Conceptualization, Data curation, Formal analysis, Software, Validation, Writing – original draft, Writing – review & editing. LZ: Formal analysis, Writing – original draft, Data curation, Software, Validation. LC: Data curation, Writing – original draft, Formal analysis, Visualization. CZ: Data curation, Writing – original draft. ZD: Funding acquisition, Project administration, Resources, Supervision, Writing – review & editing, Investigation, Writing – original draft. SL: Funding acquisition, Project administration, Resources, Supervision, Writing – review & editing, Conceptualization, Writing – original draft.
