# Supplementary material for: Causal role of immune cell phenotypes in idiopathic sudden sensorineural hearing loss: a bi-directional Mendelian randomization study
Source: Front Neurol. 2024 Apr 17;15:1368002. doi: 10.3389/fneur.2024.1368002 (PMC11061525; doi:10.3389/fneur.2024.1368002)
Supplement: Supplementary file 2 [file Data_Sheet_2.docx]

**Table.S1 Results of batch screening of immune cell phenotypes for idiopathic sudden sensorineural hearing loss (SSHL) by the IWV approach (forward MR analysis).**

| Methods | Id exposure | Id outcome | Exposure | Outcome | P value |
| --- | --- | --- | --- | --- | --- |
| MR-PRESSO | ebi-a-GCST90001430 | MVDw59 | SSHL | IgD^+^ CD38^dim^ %lymphocyte | 0.311 |
| MR-PRESSO | ebi-a-GCST90001398 | D3bYLG | SSHL | Unsw mem AC | 0.279 |
| MR-PRESSO | ebi-a-GCST90001464 | M75iEr | SSHL | CD86^+^ myeloid DC AC | 0.407 |
| MR-PRESSO | ebi-a-GCST90001531 | MVDw59 | SSHL | CD33^dim^ HLA DR^-^ AC | 0.981 |
| MR-PRESSO | ebi-a-GCST90002082 | 99MAuu | SSHL | SSC-A on CD8^br^ | 0.450 |
| MR-PRESSO | ebi-a-GCST90001536 | uchPZN | SSHL | CD45RA^-^ CD4^+^ %T cell | 0.561 |
| MR-PRESSO | ebi-a-GCST90001594 | ZuD9Gw | SSHL | DP (CD4^+^CD8^+^) AC | 0.961 |

**Table.S2 Exploring the causal relationship between immune cell characteristics on SSHL (forward MR analysis).**

| Analysis | Method | N snps | OR(95%CI) | P value |
| --- | --- | --- | --- | --- |
| IgD^+^ CD38^dim^ %lymphocyte |  |  |  |  |
|  | MR Egger | 33 | 1.0374(0.9959,1.0805) | 0.087683391 |
|  | Weighted median | 33 | 1.0139(0.9657,1.0644) | 0.578904683 |
|  | Inverse variance weighted | 33 | 1.0374(1.0019,1.0742) | 0.038741503 |
|  | Simple mode | 33 | 1.0692(0.9478,1.2062) | 0.284730194 |
|  | Weighted mode | 33 | 1.0366(1.0015,1.0729) | 0.048890043 |
| Unsw mem AC |  |  |  |  |
|  | MR Egger | 18 | 0.9999(0.7821,1.2783) | 0.99934189 |
|  | Weighted median | 18 | 1.126(0.9618,1.3181) | 0.140045145 |
|  | Inverse variance weighted | 18 | 1.1212(1.004,1.2522) | 0.042327576 |
|  | Simple mode | 18 | 1.2549(0.961,1.6387) | 0.113635289 |
|  | Weighted mode | 18 | 1.2363(0.9835,1.5539) | 0.086791506 |
| CD86^+^ myeloid DC AC |  |  |  |  |
|  | MR Egger | 21 | 1.0509(0.9854,1.1208) | 0.147257572 |
|  | Weighted median | 21 | 1.0538(0.9887,1.1231) | 0.107269495 |
|  | Inverse variance weighted | 21 | 1.0602(1.0083,1.1147) | 0.022383824 |
|  | Simple mode | 21 | 1.0081(0.8864,1.1464) | 0.903764175 |
|  | Weighted mode | 21 | 1.0476(0.9951,1.1029) | 0.091483874 |
| CD33^dim^ HLA DR^-^ AC |  |  |  |  |
|  | MR Egger | 24 | 1.0295(0.9966,1.0635) | 0.093430312 |
|  | Weighted median | 24 | 1.026(0.9917,1.0616) | 0.13911108 |
|  | Inverse variance weighted | 24 | 1.0311(1.0046,1.0583) | 0.021233062 |
|  | Simple mode | 24 | 1.0635(1.0163,1.113) | 0.014153844 |
|  | Weighted mode | 24 | 1.0284(0.9968,1.0611) | 0.091933903 |
| SSC-A on CD8^br^ |  |  |  |  |
|  | MR Egger | 17 | 1.0566(0.9721,1.1484) | 0.21511949 |
|  | Weighted median | 17 | 1.0889(1.0005,1.1851) | 0.048594659 |
|  | Inverse variance weighted | 17 | 1.072(1.0028,1.1461) | 0.041200052 |
|  | Simple mode | 17 | 1.1443(0.935,1.4005) | 0.20940121 |
|  | Weighted mode | 17 | 1.087(1.0092,1.1708) | 0.04265 |
| CD45RA^-^ CD4^+^ %T cell |  |  |  |  |
|  | MR Egger | 25 | 1.0284(1.0017,1.0559) | 0.048471669 |
|  | Weighted median | 25 | 1.0202(0.9892,1.0521) | 0.203742316 |
|  | Inverse variance weighted | 25 | 1.0267(1.0036,1.0503) | 0.023202117 |
|  | Simple mode | 25 | 1.0315(0.9659,1.1016) | 0.36416626 |
|  | Weighted mode | 25 | 1.0291(1.0019,1.057) | 0.046383072 |
| DP (CD4^+^CD8^+^) AC |  |  |  |  |
|  | MR Egger | 18 | 1.0749(0.9114,1.2677) | 0.403757918 |
|  | Weighted median | 18 | 1.1003(0.9732,1.2439) | 0.12686778 |
|  | Inverse variance weighted | 18 | 1.1056(1.011,1.2091) | 0.02779865 |
|  | Simple mode | 18 | 1.1265(0.9306,1.3637) | 0.23826853 |
|  | Weighted mode | 18 | 1.0879(0.9498,1.2462) | 0.240513795 |

**Table.S3 Sensitivity testing of MR analysis using the MR-PRESSO method (forward MR analysis).**

| Methods | Id exposure | Id outcome | Exposure | Outcome | P value |
| --- | --- | --- | --- | --- | --- |
| MR-PRESSO | ebi-a-GCST90001430 | MVDw59 | SIHL | IgD^+^ CD38^dim^ %lymphocyte | 0.311 |
| MR-PRESSO | ebi-a-GCST90001398 | D3bYLG | SIHL | Unsw mem AC | 0.279 |
| MR-PRESSO | ebi-a-GCST90001464 | M75iEr | SIHL | CD86^+^ myeloid DC AC | 0.407 |
| MR-PRESSO | ebi-a-GCST90001531 | MVDw59 | SIHL | CD33^dim^ HLA DR^-^ AC | 0.981 |
| MR-PRESSO | ebi-a-GCST90002082 | 99MAuu | SIHL | SSC-A on CD8^br^ | 0.450 |
| MR-PRESSO | ebi-a-GCST90001536 | uchPZN | SIHL | CD45RA^-^ CD4^+^ %T cell | 0.561 |
| MR-PRESSO | ebi-a-GCST90001594 | ZuD9Gw | SIHL | DP (CD4^+^CD8^+^) AC | 0.961 |
