# Supplementary material for: Causal role of immune cell phenotypes in idiopathic sudden sensorineural hearing loss: a bi-directional Mendelian randomization study
Source: Front Neurol. 2024 Apr 17;15:1368002. doi: 10.3389/fneur.2024.1368002 (PMC11061525; doi:10.3389/fneur.2024.1368002)
Supplement: Supplementary file 3 [file Data_Sheet_3.docx]

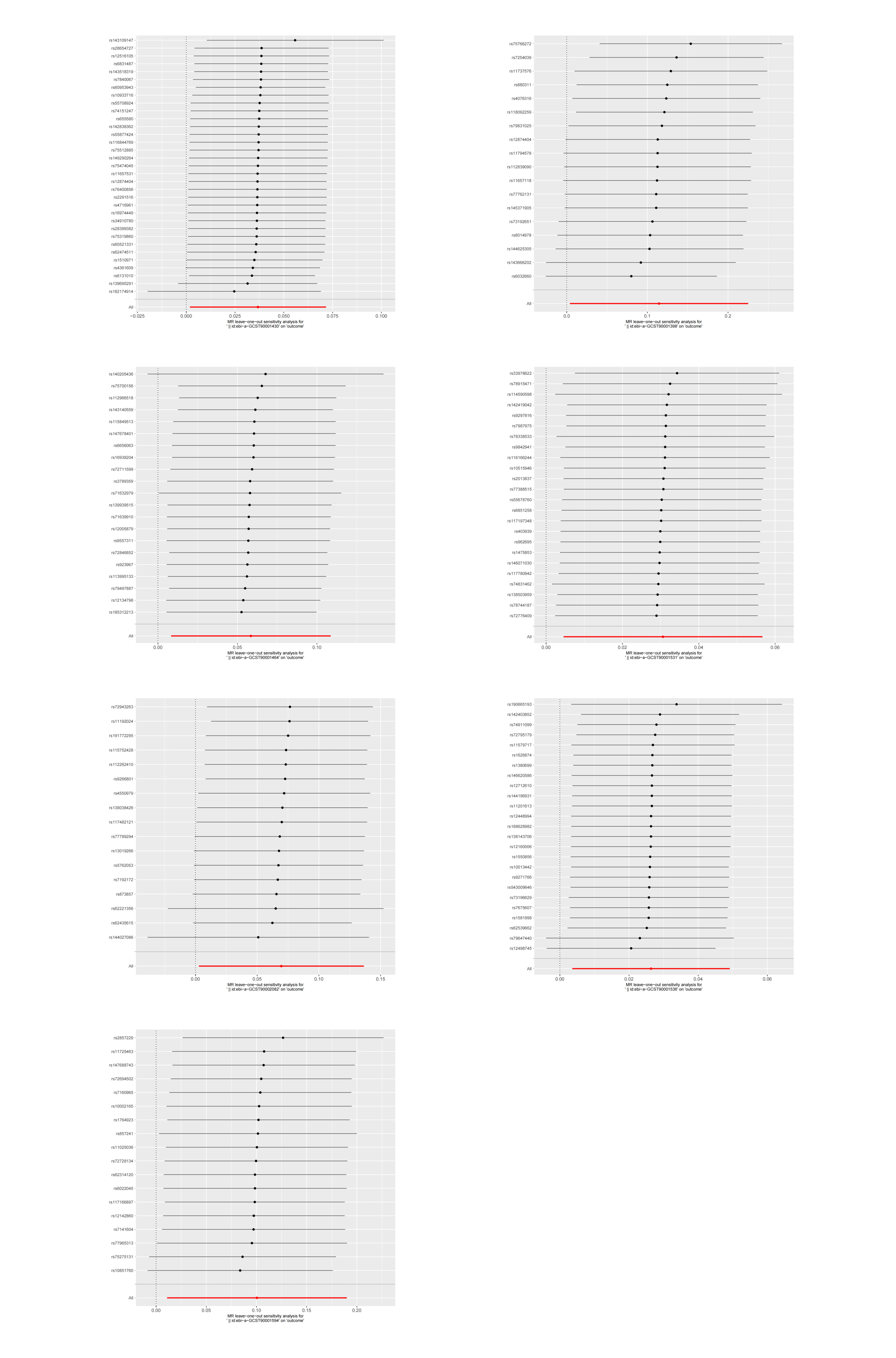


Figure.S1 Leave-one-out plots for the causal relationship between immune cell phenotype and idiopathic sudden sensorineural hearing loss (SSHL).


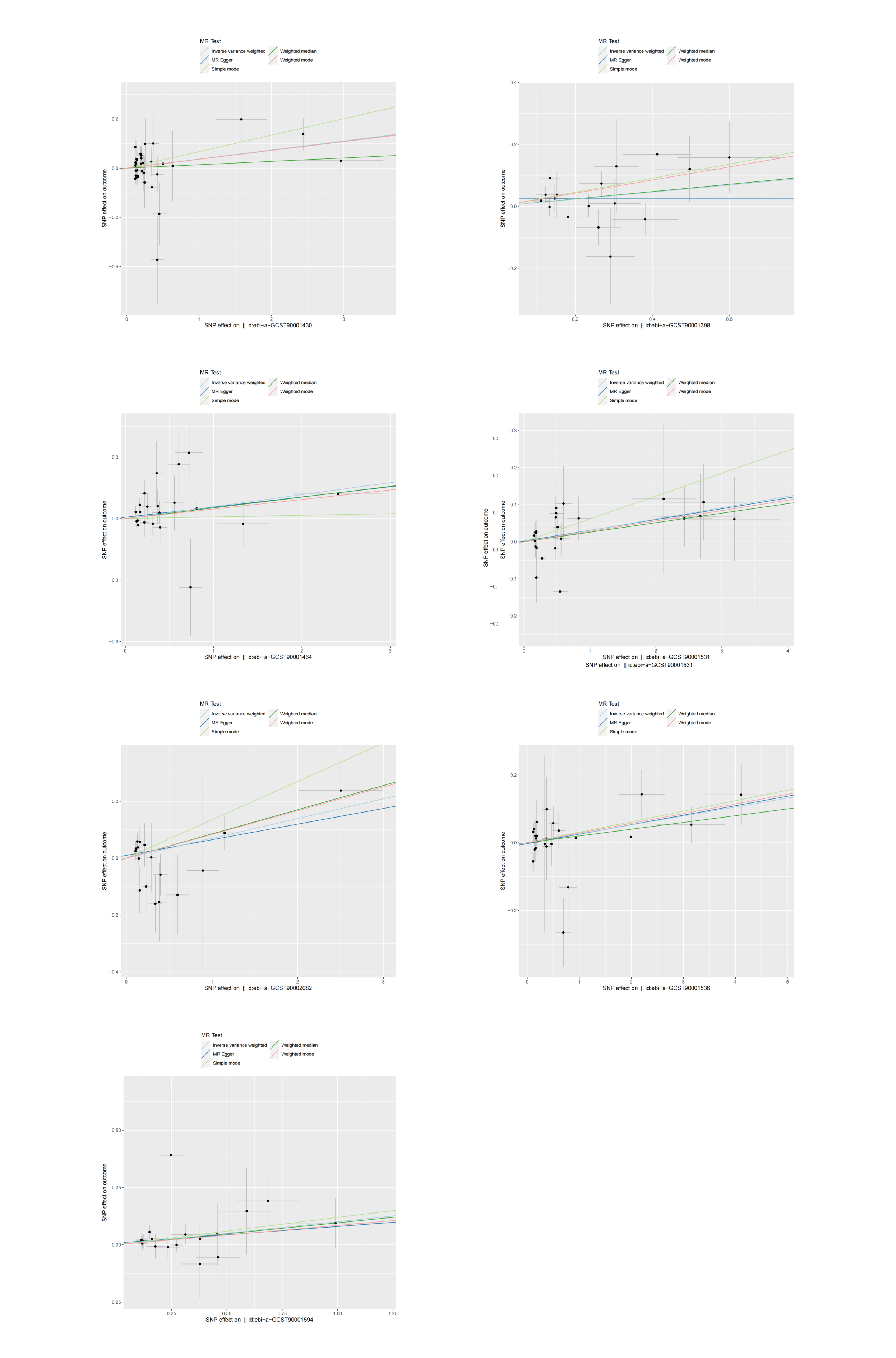


Figure.S2 Scatter plot of causal relationship between immune cell phenotype and SSHL.


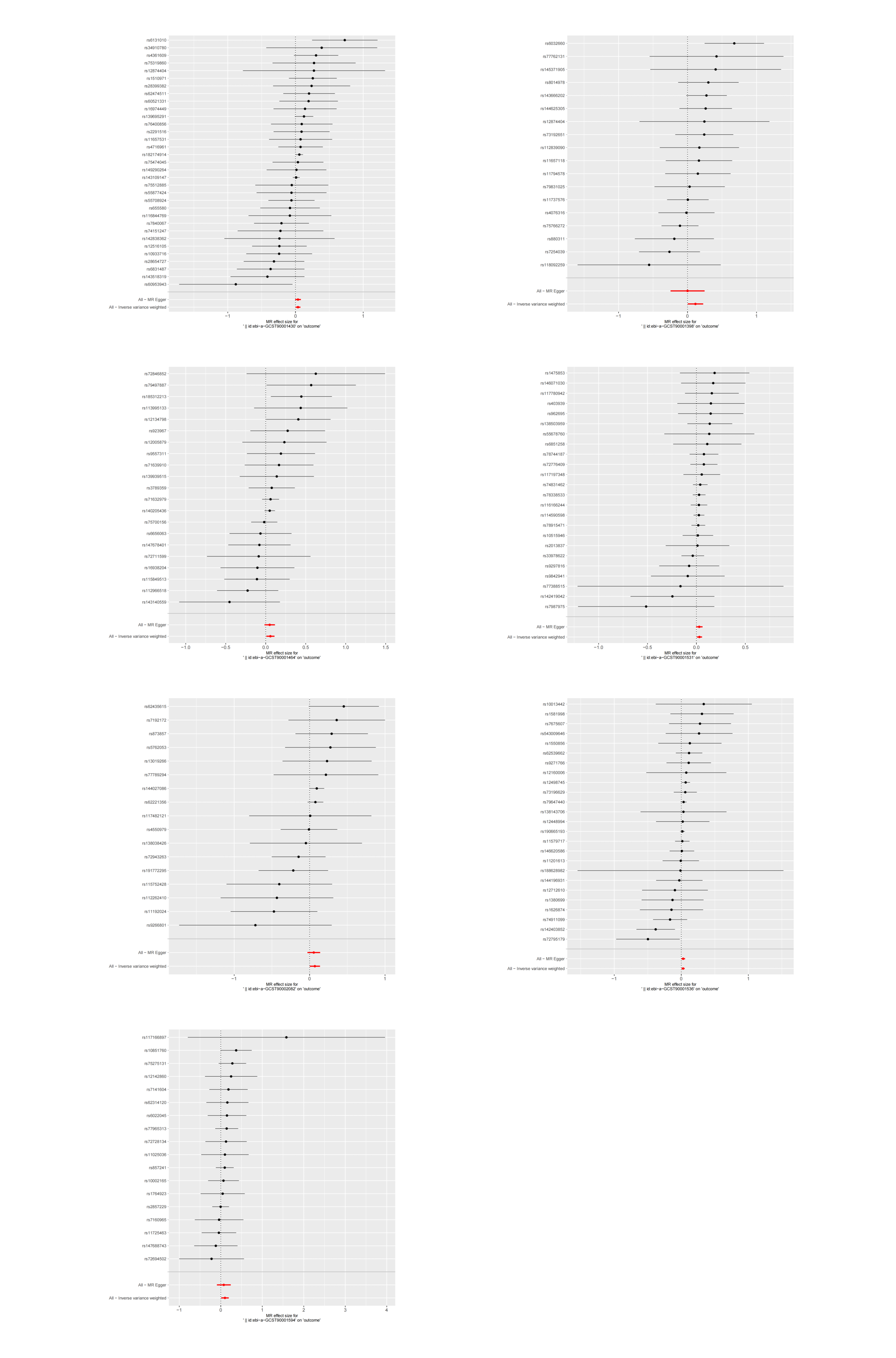


Figure.S3 Forest plot of MR effect size for causal relationship between immune phenotype and SSHL.


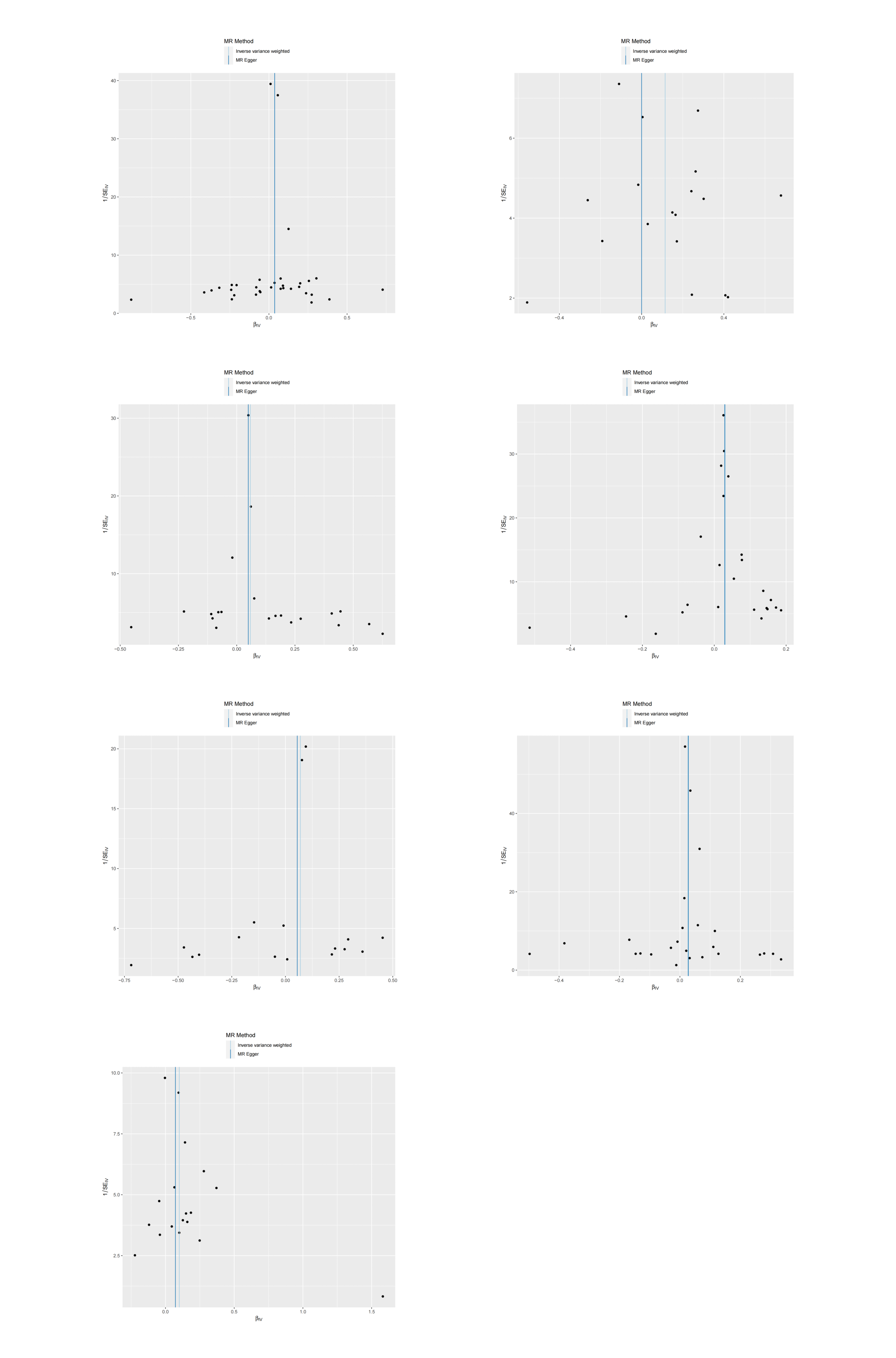


Figure.4 Funnel plot for causal relationship between immune cell phenotype and SSHL.
